# Supplementary material for: Enhancing recovery from gut microbiome dysbiosis and alleviating DSS-induced colitis in mice with a consortium of rare short-chain fatty acid-producing bacteria
Source: Gut Microbes. 2024 Jul 29;16(1):2382324. doi: 10.1080/19490976.2024.2382324 (PMC11290756; doi:10.1080/19490976.2024.2382324)
Supplement: Supplemental Material [file KGMI_A_2382324_SM7110.zip › Low_abundance_Supplementary_clean.docx]

Supplementary Material

## Supplementary Figures


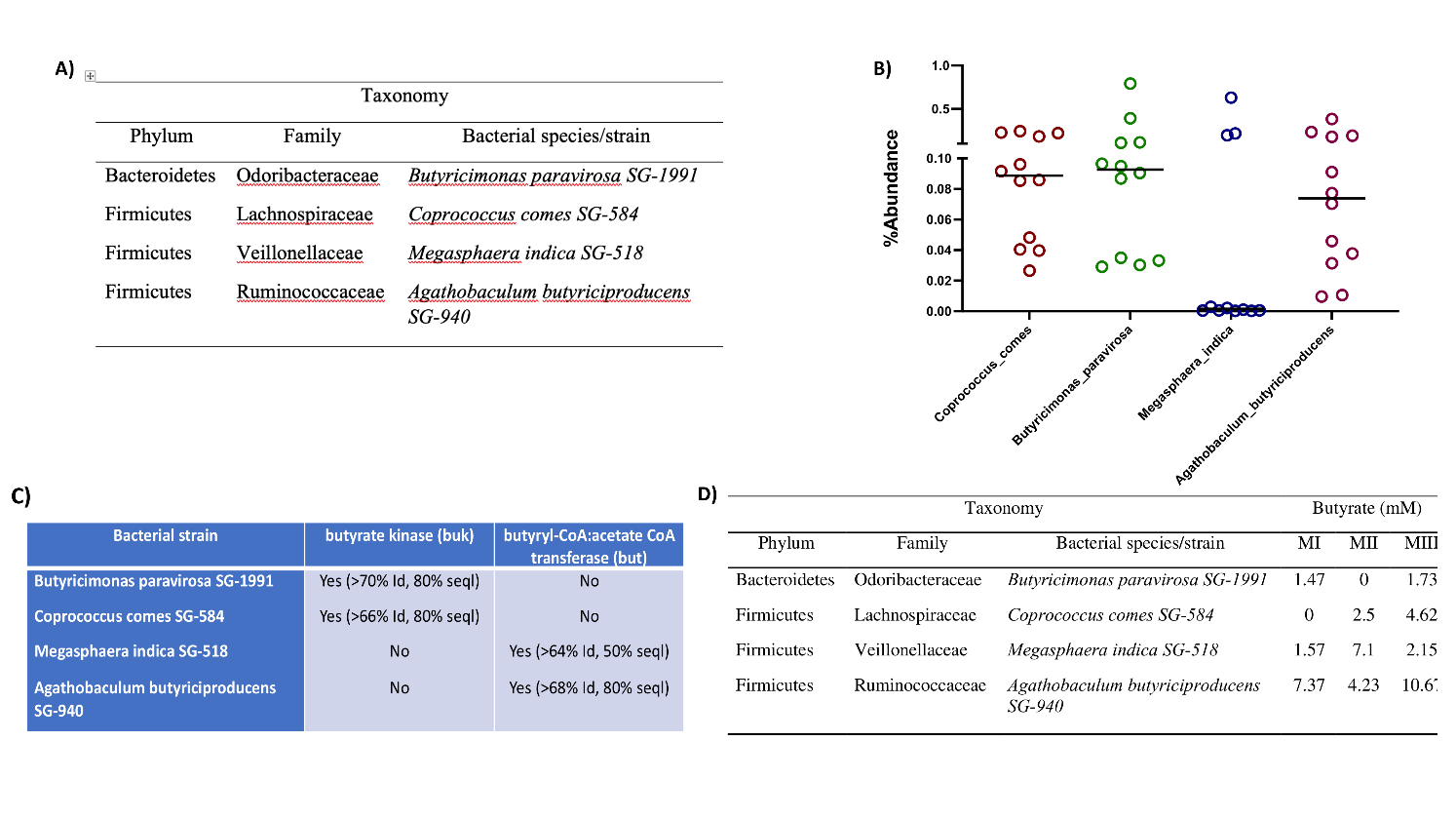
**Supplementary Figure 1.** **SC-4 species showed the ability to produce butyrate both genotypically and phenotypically. (A)** Table showing the phylum, and family to which *Coprococcus comes*, *Agathobaculum butyriciproducens, Megasphaera indica,* and *Butyricimonas paravirosa* (SC-4) species belong. **(B)** Percentage abundance of SC-4 species in donor fecal sample from which it was isolated previously. **(C)** Table showing the presence/absence and percentage identity for buk and but genes in the genome of SC-4 species. **(D)** The concentration of butyrate produced by SC-4 strains invitro in different media conditions. (MI- mBHI supplemented with Inulin, MII-mBHI, MIII- DSMZ’s modified Peptone Yeast extract Glucose )

**Supplementary Figure 2. SC-4 successfully colonized GF mice.** The total amount of bacteria found in **(A)** mice feces and Final Bacterial load in the **(B)** cecum post euthanasia (Day-28) was calculated by plating on PYG agar medium represented as Colony Forming Unit (CFU). Percentage abundance of *Coprococcus comes*, *Agathobaculum butyriciproducens, Megasphaera indica,* and *Butyricimonas paravirosa* (SC-4) in mice feces **(C)** 7 DPI, **(D)** 14 DPI, and **(E)** colon post euthanasia (Day-28).


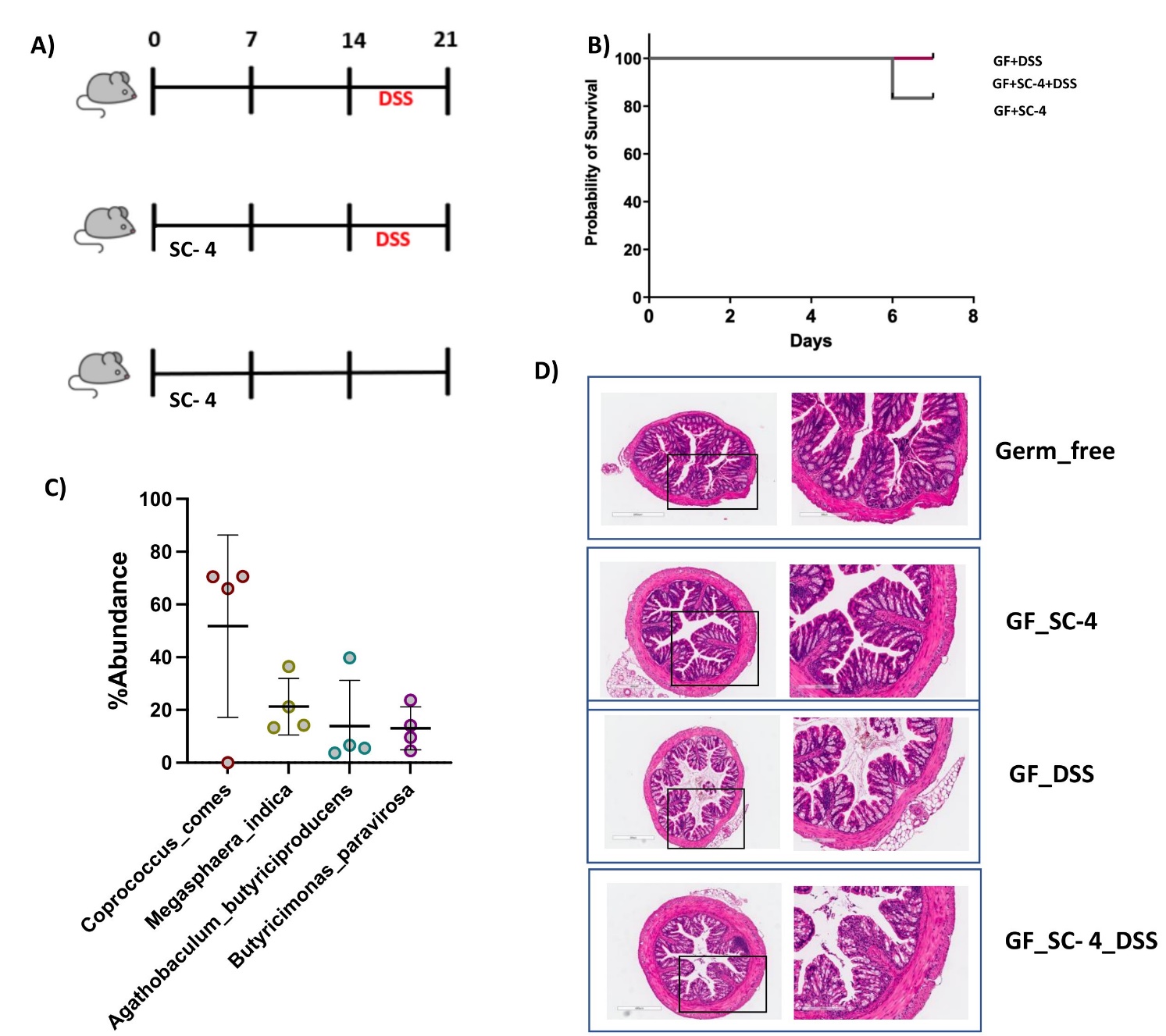


**Supplementary Figure 3. SC-4 protects against DSS-induced colitis.**  Experimental workflow for DSS-induced colitis model in **(A)** GF mice (see more details in methods). **(B)** Survival graph for GF mice induced with colitis (GF_DSS), SC-4 pre-colonized induced with colitis (GF+SC-4+DSS), or SC-4 pre-colonized only(GF+SC-4). **(C)** Percentage abundance of *Coprococcus comes*, *Agathobaculum butyriciproducens, Megasphaera indica,* and *Butyricimonas paravirosa* (SC-4) in mice colon post euthanasia (Day-21). Representative histopathological photograph showing the colon tissue cross-section after H&E staining of Germ free mice (GF) , GF mice induced with colitis (GF_DSS), GF mice gavaged with SC-4 bacteria (SF_SC-4) and SC-4 pretreated mice induced with colitis (GF_SC-4_DSS).


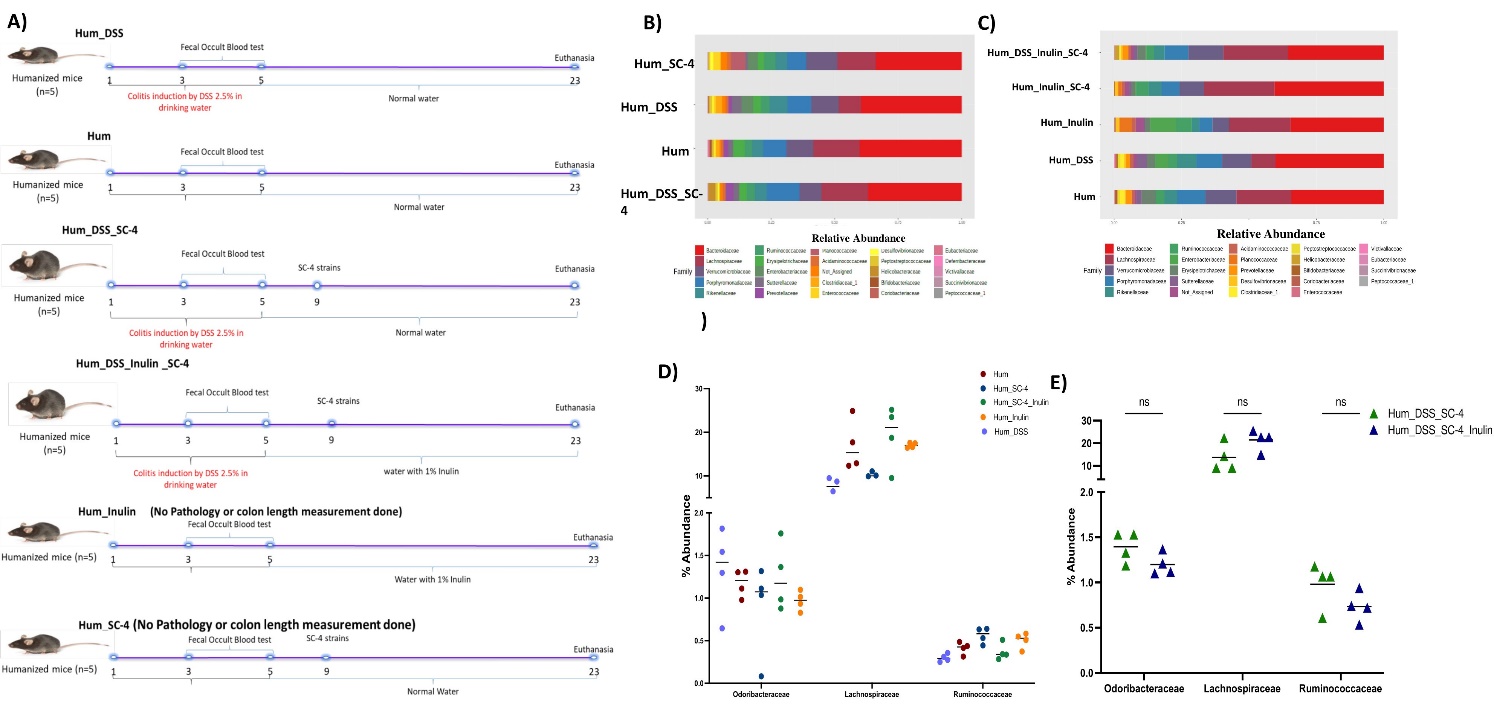


**Supplementary Figure 4. SC-4 help restore lost microbiota and help humanized mice recover from DSS-induced colitis.** Experimental outline for humanized mice based mice experiment for humanized mice with DSS-induce colitis treated with SC-4 (Hum_DSS_SC-4), Humanized mice gavage with SC_4 (Hum_SC-4), humanized mice (Hum), humanized mice induced with colitis (Hum_DSS), humanized mice treated with SC-4 with supplementation of Inulin (Hum_Inulin_SC-4), humanized mice with DSS-induce colitis treated with SC-4 with supplementation of Inulin (Hum_DSS_Inulin_SC-4) and humanized mice with supplementation of Inulin (Hum_Inulin) **(A)**. Relative abundance of bacterial community in the cecum of mice for **(B)** Hum_SC-4, Hum_DSS, Hum or **(C)** Hum_DSS_SC-4 and Hum_DSS_Inulin_SC-4, Hum_Inulin_SC-4, Hum_Inulin, Hum_DSS, or Hum. Percentage abundance of *Butyricimonas paravirosa* (Odoribacteraceae), *Coprococcus comes* (Lachnospiraceae), or *Agathobaculum butyriciproducens* (Ruminococcaceae) in mice cecum for Hum, Hum_SC-4, Hum_SC-4_Inulin, Hum_Inulin, or Hum_DSS **(D)** and Hum_DSS_SC-4 and Hum_DSS_SC-4_Inulin groups post Euthanasia.


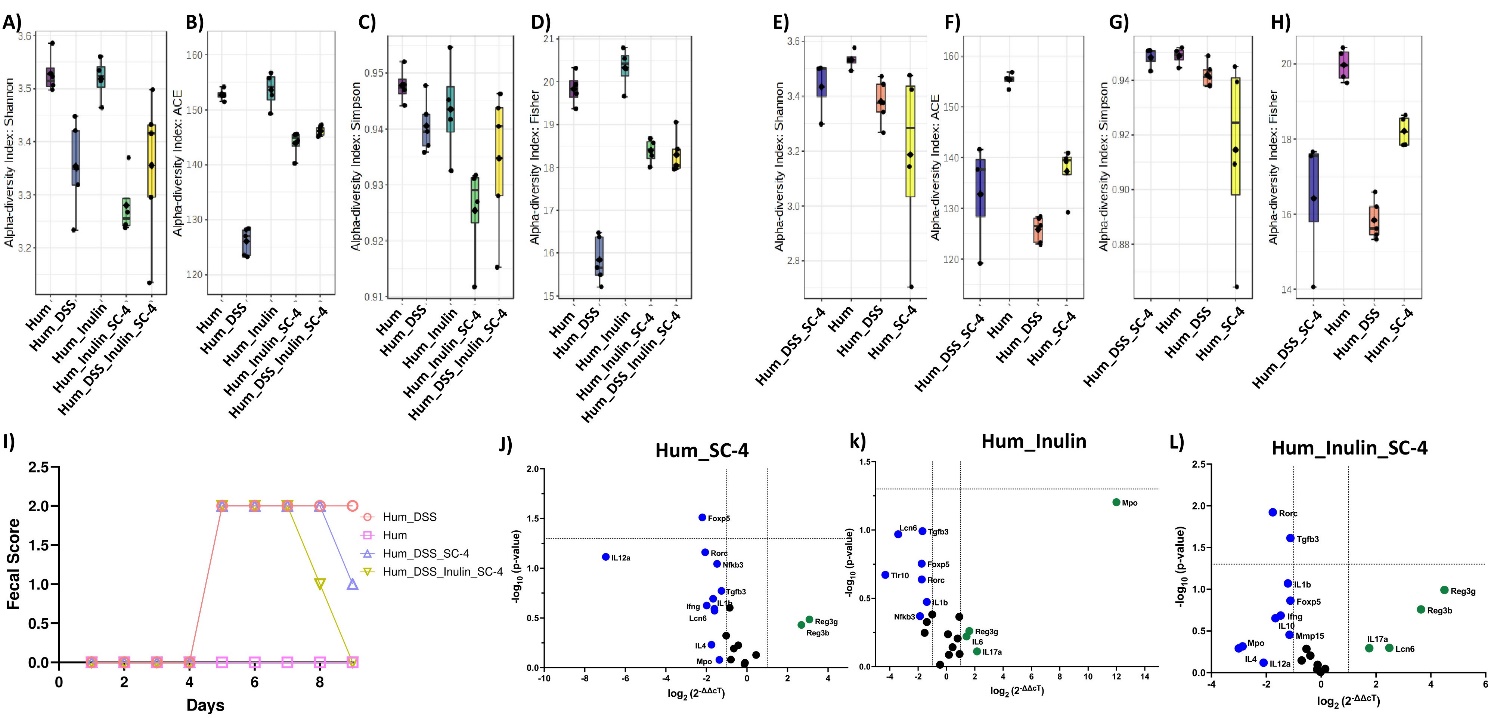


**Supplementary Figure 5. SC-4 increased the diversity of the gut microbiome in humanized mice.** Alpha diversity analysis (Shannon, ACE, Simpson, or Fisher) for humanized mice treated with SC-4 with supplementation of Inulin (Hum_Inulin_SC-4), humanized mice with DSS-induce colitis treated with SC-4 with supplementation of Inulin (Hum_DSS_Inulin_SC-4) and humanized mice with supplementation of Inulin (Hum_Inulin) **(A-D),**  humanized mice with DSS-induce colitis treated with SC-4 (Hum_DSS_SC-4), Humanized mice gavage with SC_4 (Hum_SC-4), humanized mice (Hum), humanized mice induced with colitis (Hum_DSS) **(E-H)** Fecal Score recorded for Hum_DSS, Hum, Hum_DSS_SC-4, and Hum_DSS_Inulin_SC-4 groups post DSS treatment. RT-PCR gene expression for selected 25 immune-related genes of mice colon for **(J)** Hum_SC-4, **(K)** Hum_Inulin, and **(L)** Hum_Inulin_SC-4. Green dots - overexpressed more than 2-fold change, Blue dots- represses more than 2-fold compared to Hum control (dotted lines on the x-axis represent a 2-fold increase or decrease in expression and the y-axis represents a p-value cutoff of 0.05).


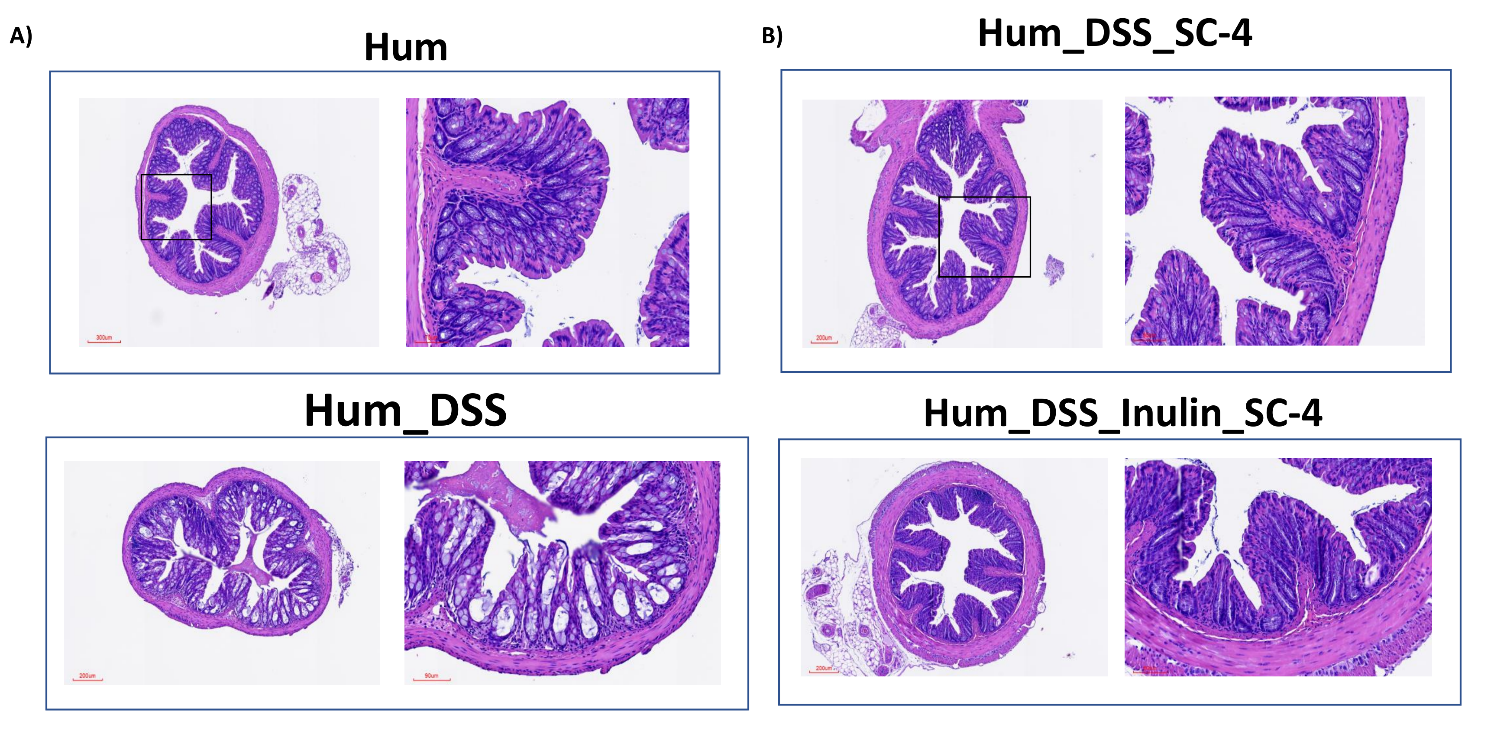


**Supplementary Figure 6. SC-4 treatment help recover colon tissue damage caused by colitis.** Representative histopathological photograph showing the colon tissue cross-section after H&E staining for humanized mice (Hum), humanized mice induced with colitis (Hum_DSS) **(A)** humanized mice with DSS-induce colitis treated with SC-4 (Hum_DSS_SC-4) and humanized mice with DSS-induce colitis treated with SC-4 with supplementation of Inulin (Hum_DSS_Inulin_SC-4) **(B) .**

## Supplementary Tables

**Supplementary Table 1** Gene names used in Rt2 profiler array gene expression analysis

| **Slno.** | **Refseq** | **Symbol** | **Description** | **RT2 Catalog** |
| --- | --- | --- | --- | --- |
| **1** | NM_013605 | Muc1 | Mucin 1, transmembrane | **PPM03608A** |
| **2** | NM_023566 | Muc2 | Mucin 2 | **PPM24739G** |
| **3** | XM_006504541 | Muc3 | Mucin 3, intestinal | **PPM25412A** |
| **4** | NM_172729 | Nod1 | Nucleotide-binding oligomerization domain containing 1 | **PPM27293C** |
| **5** | NM_145857 | Nod2 | Nucleotide-binding oligomerization domain containing 2 | **PPM33810B** |
| **6** | NM_030682 | Tlr1 | Toll-like receptor 1 | **PPM04211B** |
| **7** | NM_011905 | Tlr2 | Toll-like receptor 2 | **PPM04220B** |
| **8** | NM_021297 | Tlr4 | Toll-like receptor 4 | **PPM04207F** |
| **9** | NM_016928 | Tlr5 | Toll-like receptor 5 | **PPM04206E** |
| **10** | NM_011604 | Tlr6 | Toll-like receptor 6 | **PPM04210B** |
| **11** | NM_133211 | Tlr7 | Toll-like receptor 7 | **PPM04208A** |
| **12** | NM_133212 | Tlr8 | Toll-like receptor 8 | **PPM04213E** |
| **13** | NM_031178 | Tlr9 | Toll-like receptor 9 | **PPM04221A** |
| **14** | NM_205819 | Tlr11 | Toll-like receptor 11 | **PPM06270A** |
| **15** | NM_205823 | Tlr12 | Toll-like receptor 12 | **PPM41001A** |
| **16** | NM_205820 | Tlr13 | Toll-like receptor 13 | **PPM41490A** |
| **17** | NM_010851 | Myd88 | Myeloid differentiation primary response gene 88 | **PPM03399A** |
| **18** | NM_010554 | Il1a | Interleukin 1 alpha | **PPM03010F** |
| **19** | NM_008361 | Il1b | Interleukin 1 beta | **PPM03109F** |
| **20** | NM_008366 | Il2 | Interleukin 2 | **PPM02937C** |
| **21** | NM_021283 | Il4 | Interleukin 4 | **PPM03013F** |
| **22** | NM_010558 | Il5 | Interleukin 5 | **PPM03014F** |
| **23** | NM_001314054 | Il6 | Interleukin 6 | **PPM03015A** |
| **24** | NM_010548 | Il10 | Interleukin 10 | **PPM03017C** |
| **25** | NM_008351 | Il12a | Interleukin 12A | **PPM03019A** |
| **26** | NM_001303244 | Il12b | Interleukin 12b | **PPM03020E** |
| **27** | NM_008355 | Il13 | Interleukin 13 | **PPM03021B** |
| **28** | NM_010552 | Il17a | Interleukin 17A | **PPM03023A** |
| **29** | NM_019508 | Il17b | Interleukin 17B | **PPM03540A** |
| **30** | NM_008360 | Il18 | Interleukin 18 | **PPM03112B** |
| **31** | NM_021380 | Il20 | Interleukin 20 | **PPM03541B** |
| **32** | NM_016971 | Il22 | Interleukin 22 | **PPM05481A** |
| **33** | NM_031252 | Il23a | Interleukin 23, alpha subunit p19 | **PPM03763F** |
| **34** | NM_080729 | Il25 | Interleukin 25 | **PPM05427F** |
| **35** | NM_145636 | Il27 | Interleukin 27 | **PPM33809A** |
| **36** | NM_013693 | Tnf | Tumor necrosis factor | **PPM03113G** |
| **37** | NM_011577 | Tgfb1 | Transforming growth factor, beta 1 | **PPM02991B** |
| **38** | NM_008689 | Nfkb1 | Nuclear factor of kappa light polypeptide gene enhancer in B- cells 1, p105 | **PPM02930F** |
| **39** | NM_019408 | Nfkb2 | Nuclear factor of kappa light polypeptide gene enhancer in B- cells 2, p49/p100 | **PPM03204G** |
| **40** | NM_010507 | Ifna9 | Interferon alpha 9 | **PPM03544A** |
| **41** | NM_010510 | Ifnb1 | Interferon beta 1, fibroblast | **PPM03594C** |
| **42** | NM_008337 | Ifng | Interferon gamma | **PPM03121A** |
| **43** | NM_011259 | Reg3a | Regenerating islet-derived 3 alpha | **PPM24824A** |
| **44** | NM_011036 | Reg3b | Regenerating islet-derived 3 beta | **PPM24825A** |
| **45** | NM_011260 | Reg3g | Regenerating islet-derived 3 gamma | **PPM35204A** |
| **46** | NM_020509 | Retnla | Resistin like alpha | **PPM03005F** |
| **47** | NM_023881 | Retnlb | Resistin like beta | **PPM05091A** |
| **48** | NM_010031 | Defa1 | Defensin, alpha 1 | **PPM37773A** |
| **49** | NM_001167790 | Defa17 | Defensin, alpha, 17 | **PPM63552A** |
| **50** | NM_007843 | Defb1 | Defensin beta 1 | **PPM25297F** |
| **51** | NM_010030 | Defb2 | Defensin beta 2 | **PPM28936A** |
| **52** | NM_013756 | Defb3 | Defensin beta 3 | **PPM30650F** |
| **53** | NM_019728 | Defb4 | Defensin beta 4 | **PPM29171A** |
| **54** | NM_030734 | Defb5 | Defensin beta 5 | **PPM34137A** |
| **55** | NM_181683 | Defb37 | Defensin beta 37 | **PPM36272A** |
| **56** | NM_183038 | Defb39 | Defensin beta 39 | **PPM58991A** |
| **57** | NM_177850 | Bpi | Bactericidal permeablility increasing protein | **PPM35806A** |
| **58** | NM_001033367 | Nlrc4 | NLR family, CARD domain containing 4 | **PPM31278A** |
| **59** | NM_145827 | Nlrp3 | NLR family, pyrin domain containing 3 | **PPM29506F** |
| **60** | NM_001081389 | Nlrp6 | NLR family, pyrin domain containing 6 | **PPM26688B** |
| **61** | NM_001033431 | Nlrp12 | NLR family, pyrin domain containing 12 | **PPM37047B** |
| **62** | NM_009807 | Casp1 | Caspase 1 | **PPM02921E** |
| **63** | NM_009921 | Camp | Cathelicidin antimicrobial peptide | **PPM25023A** |
| **64** | NM_013653 | Ccl5 | Chemokine (C-C motif) ligand 5 | **PPM02960F** |
| **65** | NM_016960 | Ccl20 | Chemokine (C-C motif) ligand 20 | **PPM03142B** |
| **66** | NM_009138 | Ccl25 | Chemokine (C-C motif) ligand 25 | **PPM02972F** |
| **67** | NM_020279 | Ccl28 | Chemokine (C-C motif) ligand 28 | **PPM03603C** |
| **68** | NM_008176 | Cxcl1 | Chemokine (C-X-C motif) ligand 1 | **PPM03058C** |
| **69** | NM_018866 | Cxcl13 | Chemokine (C-X-C motif) ligand 13 | **PPM02947G** |
| **70** | NM_177850 | Bpi | Bactericidal permeablility increasing protein | **PPM35806A** |
| **71** | NM_013650 | S100a8 | S100 calcium binding protein A8 (calgranulin A) | **PPM05051F** |
| **72** | NM_009114 | S100a9 | S100 calcium binding protein A9 (calgranulin B) | **PPM05050E** |
| **73** | NM_054039 | Foxp3 | Forkhead box P3 | **PPM05497F** |
| **74** | NM_016674 | Cldn1 | Claudin 1 | **PPM05454A** |
| **75** | NM_008756 | Ocln | Occludin | **PPM05314A** |
| **76** | NM_009864 | Cdh1 | Cadherin 1 | **PPM03652F** |
| **77** | NM_009386 | Tjp1 | Tight junction protein 1 | **PPM25091A** |
| **78** | NM_007980 | Fabp2 | Fatty acid binding protein 2, intestinal | **PPM27271A** |
| **79** | NM_009196 | Slc16a1 | Solute carrier family 16 (monocarboxylic acid transporters), member 1 | **PPM25515A** |
| **80** | NM_145423 | Slc5a8 | Solute carrier family 5 (iodide transporter), member 8 | **PPM29969B** |
| **81** | NM_146187 | Ffar2 | Free fatty acid receptor 2 | **PPM04863A** |
| **82** | NM_001033316 | Ffar3 | Free fatty acid receptor 3 | **PPM59038A** |
| **83** | NM_030701 | Niacr1 | Niacin receptor 1 | **PPM03781A** |
| **84** | NM_011038 | Pax4 | Paired box gene 4 | **PPM05533A** |
| **85** | NM_013627 | Pax6 | Paired box gene 6 | **PPM04498B** |
| **86** | NM_013685 | Tcf4 | Transcription factor 4 | **PPM05459C** |
| **87** | NM_008100 | Gcg | Glucagon | **PPM04763G** |
| **88** | NM_011831 | Insl5 | Insulin-like 5 | **PPM30485A** |
| **89** | NM_145435 | Pyy | Peptide YY | **PPM29314B** |

**Supplementary Table 2** Primer details of the custom-made panel of mice immune genes.

| **Slno.** | **Refseq** | **Symbol** | **Description** | **RT2 Catalog** |
| --- | --- | --- | --- | --- |
| **1** | NM_013605 | Muc1 | Mucin 1, transmembrane | **PPM03608A** |
| **2** | NM_023566 | Muc2 | Mucin 2 | **PPM24739G** |
| **3** | XM_006504541 | Muc3 | Mucin 3, intestinal | **PPM25412A** |
| **4** | NM_172729 | Nod1 | Nucleotide-binding oligomerization domain containing 1 | **PPM27293C** |
| **5** | NM_145857 | Nod2 | Nucleotide-binding oligomerization domain containing 2 | **PPM33810B** |
| **6** | NM_030682 | Tlr1 | Toll-like receptor 1 | **PPM04211B** |
| **7** | NM_011905 | Tlr2 | Toll-like receptor 2 | **PPM04220B** |
| **8** | NM_021297 | Tlr4 | Toll-like receptor 4 | **PPM04207F** |
| **9** | NM_016928 | Tlr5 | Toll-like receptor 5 | **PPM04206E** |
| **10** | NM_011604 | Tlr6 | Toll-like receptor 6 | **PPM04210B** |
| **11** | NM_133211 | Tlr7 | Toll-like receptor 7 | **PPM04208A** |
| **12** | NM_133212 | Tlr8 | Toll-like receptor 8 | **PPM04213E** |
| **13** | NM_031178 | Tlr9 | Toll-like receptor 9 | **PPM04221A** |
| **14** | NM_205819 | Tlr11 | Toll-like receptor 11 | **PPM06270A** |
| **15** | NM_205823 | Tlr12 | Toll-like receptor 12 | **PPM41001A** |
| **16** | NM_205820 | Tlr13 | Toll-like receptor 13 | **PPM41490A** |
| **17** | NM_010851 | Myd88 | Myeloid differentiation primary response gene 88 | **PPM03399A** |
| **18** | NM_010554 | Il1a | Interleukin 1 alpha | **PPM03010F** |
| **19** | NM_008361 | Il1b | Interleukin 1 beta | **PPM03109F** |
| **20** | NM_008366 | Il2 | Interleukin 2 | **PPM02937C** |
| **21** | NM_021283 | Il4 | Interleukin 4 | **PPM03013F** |
| **22** | NM_010558 | Il5 | Interleukin 5 | **PPM03014F** |
| **23** | NM_001314054 | Il6 | Interleukin 6 | **PPM03015A** |
| **24** | NM_010548 | Il10 | Interleukin 10 | **PPM03017C** |
| **25** | NM_008351 | Il12a | Interleukin 12A | **PPM03019A** |
| **26** | NM_001303244 | Il12b | Interleukin 12b | **PPM03020E** |
| **27** | NM_008355 | Il13 | Interleukin 13 | **PPM03021B** |
| **28** | NM_010552 | Il17a | Interleukin 17A | **PPM03023A** |
| **29** | NM_019508 | Il17b | Interleukin 17B | **PPM03540A** |
| **30** | NM_008360 | Il18 | Interleukin 18 | **PPM03112B** |
| **31** | NM_021380 | Il20 | Interleukin 20 | **PPM03541B** |
| **32** | NM_016971 | Il22 | Interleukin 22 | **PPM05481A** |
| **33** | NM_031252 | Il23a | Interleukin 23, alpha subunit p19 | **PPM03763F** |
| **34** | NM_080729 | Il25 | Interleukin 25 | **PPM05427F** |
| **35** | NM_145636 | Il27 | Interleukin 27 | **PPM33809A** |
| **36** | NM_013693 | Tnf | Tumor necrosis factor | **PPM03113G** |
| **37** | NM_011577 | Tgfb1 | Transforming growth factor, beta 1 | **PPM02991B** |
| **38** | NM_008689 | Nfkb1 | Nuclear factor of kappa light polypeptide gene enhancer in B- cells 1, p105 | **PPM02930F** |
| **39** | NM_019408 | Nfkb2 | Nuclear factor of kappa light polypeptide gene enhancer in B- cells 2, p49/p100 | **PPM03204G** |
| **40** | NM_010507 | Ifna9 | Interferon alpha 9 | **PPM03544A** |
| **41** | NM_010510 | Ifnb1 | Interferon beta 1, fibroblast | **PPM03594C** |
| **42** | NM_008337 | Ifng | Interferon gamma | **PPM03121A** |
| **43** | NM_011259 | Reg3a | Regenerating islet-derived 3 alpha | **PPM24824A** |
| **44** | NM_011036 | Reg3b | Regenerating islet-derived 3 beta | **PPM24825A** |
| **45** | NM_011260 | Reg3g | Regenerating islet-derived 3 gamma | **PPM35204A** |
| **46** | NM_020509 | Retnla | Resistin like alpha | **PPM03005F** |
| **47** | NM_023881 | Retnlb | Resistin like beta | **PPM05091A** |
| **48** | NM_010031 | Defa1 | Defensin, alpha 1 | **PPM37773A** |
| **49** | NM_001167790 | Defa17 | Defensin, alpha, 17 | **PPM63552A** |
| **50** | NM_007843 | Defb1 | Defensin beta 1 | **PPM25297F** |
| **51** | NM_010030 | Defb2 | Defensin beta 2 | **PPM28936A** |
| **52** | NM_013756 | Defb3 | Defensin beta 3 | **PPM30650F** |
| **53** | NM_019728 | Defb4 | Defensin beta 4 | **PPM29171A** |
| **54** | NM_030734 | Defb5 | Defensin beta 5 | **PPM34137A** |
| **55** | NM_181683 | Defb37 | Defensin beta 37 | **PPM36272A** |
| **56** | NM_183038 | Defb39 | Defensin beta 39 | **PPM58991A** |
| **57** | NM_177850 | Bpi | Bactericidal permeablility increasing protein | **PPM35806A** |
| **58** | NM_001033367 | Nlrc4 | NLR family, CARD domain containing 4 | **PPM31278A** |
| **59** | NM_145827 | Nlrp3 | NLR family, pyrin domain containing 3 | **PPM29506F** |
| **60** | NM_001081389 | Nlrp6 | NLR family, pyrin domain containing 6 | **PPM26688B** |
| **61** | NM_001033431 | Nlrp12 | NLR family, pyrin domain containing 12 | **PPM37047B** |
| **62** | NM_009807 | Casp1 | Caspase 1 | **PPM02921E** |
| **63** | NM_009921 | Camp | Cathelicidin antimicrobial peptide | **PPM25023A** |
| **64** | NM_013653 | Ccl5 | Chemokine (C-C motif) ligand 5 | **PPM02960F** |
| **65** | NM_016960 | Ccl20 | Chemokine (C-C motif) ligand 20 | **PPM03142B** |
| **66** | NM_009138 | Ccl25 | Chemokine (C-C motif) ligand 25 | **PPM02972F** |
| **67** | NM_020279 | Ccl28 | Chemokine (C-C motif) ligand 28 | **PPM03603C** |
| **68** | NM_008176 | Cxcl1 | Chemokine (C-X-C motif) ligand 1 | **PPM03058C** |
| **69** | NM_018866 | Cxcl13 | Chemokine (C-X-C motif) ligand 13 | **PPM02947G** |
| **70** | NM_177850 | Bpi | Bactericidal permeablility increasing protein | **PPM35806A** |
| **71** | NM_013650 | S100a8 | S100 calcium binding protein A8 (calgranulin A) | **PPM05051F** |
| **72** | NM_009114 | S100a9 | S100 calcium binding protein A9 (calgranulin B) | **PPM05050E** |
| **73** | NM_054039 | Foxp3 | Forkhead box P3 | **PPM05497F** |
| **74** | NM_016674 | Cldn1 | Claudin 1 | **PPM05454A** |
| **75** | NM_008756 | Ocln | Occludin | **PPM05314A** |
| **76** | NM_009864 | Cdh1 | Cadherin 1 | **PPM03652F** |
| **77** | NM_009386 | Tjp1 | Tight junction protein 1 | **PPM25091A** |
| **78** | NM_007980 | Fabp2 | Fatty acid binding protein 2, intestinal | **PPM27271A** |
| **79** | NM_009196 | Slc16a1 | Solute carrier family 16 (monocarboxylic acid transporters), member 1 | **PPM25515A** |
| **80** | NM_145423 | Slc5a8 | Solute carrier family 5 (iodide transporter), member 8 | **PPM29969B** |
| **81** | NM_146187 | Ffar2 | Free fatty acid receptor 2 | **PPM04863A** |
| **82** | NM_001033316 | Ffar3 | Free fatty acid receptor 3 | **PPM59038A** |
| **83** | NM_030701 | Niacr1 | Niacin receptor 1 | **PPM03781A** |
| **84** | NM_011038 | Pax4 | Paired box gene 4 | **PPM05533A** |
| **85** | NM_013627 | Pax6 | Paired box gene 6 | **PPM04498B** |
| **86** | NM_013685 | Tcf4 | Transcription factor 4 | **PPM05459C** |
| **87** | NM_008100 | Gcg | Glucagon | **PPM04763G** |
| **88** | NM_011831 | Insl5 | Insulin-like 5 | **PPM30485A** |
| **89** | NM_145435 | Pyy | Peptide YY | **PPM29314B** |

**Supplementary Table 3.** List of antibodies with fluorochrome used for staining the immune cells under panel 1&2.

| **Fluorochrome** | **Cell type** | **Antigen** | **Clone** | **Product** |
| --- | --- | --- | --- | --- |
| **Panel -1** | | | | |
| FITC | Lymphocytes total | CD45 | 30-F11 | CD45 Monoclonal Antibody (30-F11), FITC, eBioscience |
| PE | B cells | CD19 | eBio1D3 (1D3 | CD19 Monoclonal Antibody (eBio ID3(ID3)), PE, eBioscience |
| PE-Cyanine7 | Tgd | TCR gamma/delta | eBioGL3 (GL-3,GL3) | TCR gamma/delta Monoclonal Antibody (eBioGL3(GL-3,GL3)), PE-Cyanine7, eBioscience |
| APC | ILC3 | ROR gamma (t) | AFKJS-9 | ROR gamma (t) Monoclonal Antibody (AFKJS-9), APC, eBioscience |
| Alexa Fluor 700 | Tab | TCR beta | H57-597 | TCR beta Monoclonal Antibody (H57-597), Alexa Fluor 700, ebioscience |
| LIVE/DEAD Fixable Near-IR Dead Cell Stain | Live cells |  |  | LIVE/DEAD Fixable Near-IR Dead cell Stain Kit, for 633 or 635 nm excitation |
| **Panel -2** | | | | |
| FITC | Lymphocytes total | CD45 | 30-F11 | CD45 Monoclonal Antibody (30-F11), FITC, eBioscience |
| PE | CD8 cells | CD8 | 53-6.7 | CD19 Monoclonal Antibody (53-6.7), PE, eBioscience |
| PE-Cyanine5.5 | CD4 cells | CD45 | RM4-5 | CD4 Monoclonal Antibody (RM4-5),PE-Cyanine5.5, eBioscience |
| PE-Cyanine7 | Treg | FOXP-3 | FJK-16s | FOXP3 Monoclonal Antibody (FJK-16s),PE-Cyanine7, eBioscience |
| APC | Th17 | ROR gamma (t) | AFKJS-9 | ROR gamma (t) Monoclonal Antibody (AFKJS-9), APC, eBioscience |
| Alexa Fluor 700 | Tab | TCR beta | H57-597 | TCR beta Monoclonal Antibody (H57-597), Alexa Fluor 700, ebioscience |
| LIVE/DEAD Fixable Near-IR Dead Cell Stain | Live cells |  |  | LIVE/DEAD Fixable Near-IR Dead cell Stain Kit, for 633 or 635 nm excitation |
